# Overlooked evidence for semantic compositionality and signal reduction in wild chimpanzees (*Pan troglodytes*)

**DOI:** 10.1007/s10071-021-01584-3

**Published:** 2021-11-25

**Authors:** Petar Gabrić

**Affiliations:** 1 Institute for German Linguistics, Philipps University of Marburg, Pilgrimstein 16, 35032 Marburg, Germany; 2 Department of Psychiatry and Psychotherapy, Philipps University of Marburg, Marburg, Germany

**Keywords:** Chimpanzee communication, Animal communication, Language evolution, Semantic compositionality, Non-vocal communication

## Abstract

Recent discoveries of semantic compositionality in Japanese tits have enlivened the discussions on the presence of this phenomenon in wild animal communication. Data on semantic compositionality in wild apes are lacking, even though language experiments with captive apes have demonstrated they are capable of semantic compositionality. In this paper, I revisit the study by Boesch (Hum. Evol. 6:81–89, 1991) who investigated drumming sequences by an alpha male in a chimpanzee (*Pan troglodytes*) community in the Taï National Park, Côte d’Ivoire. A reanalysis of the data reveals that the alpha male produced semantically compositional combined messages of travel direction change and resting period initiation. Unlike the Japanese tits, the elements of the compositional expression were not simply juxtaposed but displayed structural reduction, while one of the two elements in the expression coded the meanings of both elements. These processes show relative resemblance to blending and fusion in human languages. Also unlike the tits, the elements of the compositional expression did not have a fixed order, although there was a fixed distribution of drumming events across the trees used for drumming. Because the elements of the expression appear to carry verb-like meanings, the compositional expression also resembles simple verb-verb constructions and short paratactic combinations of two clauses found across languages. In conclusion, the reanalysis suggests that semantic compositionality and phenomena resembling paratactic combinations of two clauses might have been present in the communication of the last common ancestor of chimpanzees and humans, not necessarily in the vocal modality.

## Introduction

Language evolution is a hotly debated topic. However, our ability to investigate it is severely limited due to a multitude of factors, most prominently the lack of valid methods and techniques. One approach has been to study the communication of non-human animals and, specifically, of our closest living relatives—the common chimpanzees and bonobos. Discoveries of elements of what is today considered to be linguistic communication in wild members of the *Pan* genus would greatly affect how we understand language evolution, as well as how we understand linguistic communication in general. In the present paper, I revisit the study by Boesch (1991) who investigated drumming behavior by an alpha male in a community of wild chimpanzees (*Pan troglodytes*), with the aim of demonstrating for the first time that one of the fundamental linguistic phenomena—the ability to combine meaningful elements—is present in wild chimpanzee communication. In the introductory part of the paper, I first define semantic compositionality and emphasize its vital role in linguistic communication. Following this, I draw on a previous study that reported that a wild songbird displays semantic compositionality in their communication. Then I shortly review the current evidence, or lack of it, for semantic compositionality in wild primates and, specifically, wild apes, arguing that this is somewhat unexpected given that language experiments with captive apes have revealed they are capable of semantic compositionality.

### Semantic compositionality

Language is considered to be a human-specific trait. However, our understanding of precisely which aspects of language should be defined as human-specific remains poor. On the other hand, research on wild animal communication from the last several decades suggests that analogical phenomena to those observed in language can be described in wild animal communication as well.

Specifically, recent research has suggested that one of the fundamental features of language—semantic compositionality—might be extant in some wild animal communication systems. Semantic compositionality refers to the communicational capacity to combine structures and their meanings into sequences with “derived” meanings, with the sequence’s meaning being a function of the meanings of its parts and the rule(s)^1^ applied to arrange the parts^2^ (Hackl 2013; Hurford 2012; Kemp 2013; Löbner 2013; Saeed 2016). For example, a speaker of English uttering *John broke the glass.* does not only intend to convey a message about an event involving john, breaking, and glass^3^. By arranging the words in the given manner, the different parts of the utterance are assigned, among other functions, different semantic roles: *John* is the agent (the one performing an action), *broke* is the predicate (in this case, the action), while *the glass* is the patient (the object on which the action is being performed). Only by combining these elements in a sentence using specific rules (such as word order in this case) can the compositional meaning of an agent-action-patient event be transparently coded and transmitted. Otherwise, and in this particular context, the receiver could never be able to tell who did what to whom and the relative functionality of such a communicational system would be questionable (cf. Gabrić 2021a). This is why semantic compositionality is fundamental to language and why it may have been present in the earliest stages of language evolution (Gabrić 2019, 2021a, b, c, d; Gabrić et al. 2021; Hurford 2012; Jackendoff and Wittenberg 2014; Progovac 2015, 2016, 2019). In one view of language functioning, any combination of at least two elements forming an utterance (in that case, sentence) follows at least some rule, implying that semantic compositionality is intrinsic to linguistic communication.

### Semantic compositionality in wild animal communication

Research has thus far demonstrated that some wild birds’ and primates’ vocalizations can be characterized as having a lexical or word-like character, in the sense that they denote concepts (e.g., predators in alarm calls or foods in food calls) similarly to how words in human languages denote (i.e., mean, map to) certain concepts (Gill and Bierema 2013; Macedonia and Evans 1993; Townsend and Manser 2013; Zuberbühler 2009). This has been reported for different taxa, including, for example, chickens (*Gallus*; Evans et al. 1993; Karakashian et al. 1988), trumpeters (*Psophia*; Seddon et al. 2002), tits (*Paridae*; Ha et al. 2020; Haftorn 2000; Suzuki 2011, 2012, 2014, 2015, 2016a, b, 2018, 2019, 2020, 2021), titi monkeys (*Callicebinae*; Cäsar et al. 2012; Schlenker et al. 2017), capuchins (*Cebinae*; Digweed et al. 2005), macaques (*Macaca*; Brumm et al. 2004), *Chlorocebi* (including vervets and green monkeys; Cheney and Seyfarth 1990; Fischer 2020; Lyn and Christopher 2020; Seyfarth et al. 1980a, b; Snowdon 2020; Struhsaker 1967), guenons (*Cercopithecus*; Arnold et al. 2008, 2010; Arnold and Zuberbühler 2006b; Zuberbühler et al. 1999), and chimpanzees (*Pan*; Clay and Zuberbühler 2009; Crockford and Boesch 2003; Slocombe and Zuberbühler 2005; Uhlenbroek 1996).

Furthermore, numerous studies have suggested that some communication systems of wild and some domestic bird, whale, and primate taxa exhibit syntactic properties, i.e., rules for combining different structures or structure types (Berwick et al. 2011, 2012; Griesser et al. 2018; Steinert-Threlkeld 2016; Suzuki et al. 2018, 2019; ten Cate and Okanoya 2012), including, for example, Australo-Papuan babblers (*Pomatostomidae*; Engesser et al. 2019), tanagers (*Thraupidae*; Fandiño-Mariño and Vielliard 2004), hummingbirds (*Trochilidae*; Ficken et al. 2000), tits (*Paridae*; Hailman et al. 1985), chickadees (*Poecile*; Freeberg 2008; Freeberg and Lucas 2012), true finches (*Fringillidae*; Riebel and Slater 2003), New World sparrows (*Passerellidae*; Rose et al. 2004), estrildid finches (*Estrildidae*; Abe and Watanabe 2011; Beckers et al. 2012; Honda and Okanoya 1999; Katahira et al. 2007; Leonardo 2002; Sturdy et al. 1999; van Heijningen et al. 2009), nightingales and relatives (*Luscinia*; Hultsch and Todt 1989; Todt and Hultsch 1996, 1998), starlings (*Sturnidae*; Gentner et al. 2006), gulls (*Laridae*; Beer 1976), bats (*Chiroptera*; Bohn et al. 2013), mice (Chabout et al. 2015), mongooses (*Herpestidae*; Fitch 2012), humpback whales (*Megaptera novaeangliae*; Mercado et al. 2005; Payne and McVay 1971; Suzuki et al. 2006), tamarins (*Saguinus*; Fitch 2012), capuchins (*Cebinae*; Robinson 1984), and gibbons (*Hylobatidae*; Clarke et al. 2006; Haimoff 1985; Inoue et al. 2017, 2020a, b; Terleph et al. 2018). Hurford (2012: 97) comments: “Despite serious underexploitation of combinatoriality, […] whalesong and much birdsong exhibit a hierarchically layered structure formally similar to the hierarchical structure of human syntax.” However, these behaviors do not qualify for semantic compositionality because, according to available data, the elements being combined do not map to individual meanings (Hurford 2012).

Data on semantic compositionality in wild animal communication are sparse. Recently, Suzuki et al. (2016) reported from an experimental study that the Japanese tit (*Parus minor*), a passerine bird, can combine different combinations of notes to express a compositional meaning. The note combination *ABC*—typically eliciting a scan-for-danger response in the receiver in a predatory context—followed by *D*—typically eliciting an approach-the-caller response in the receiver in a non-predatory context—unitarily conveys the messages scan for danger and approach the caller in a predatory context. In other words, after hearing *ABC-D*, the receivers would display the scan-for-danger (horizontal head scanning) and approach-the-caller responses at similar rates as they would after hearing solely *ABC* and *D*, respectively. However, the artificial combination *D-ABC* elicited significantly fewer behavioral responses in the receivers, tentatively indicating the existence of some rule(s) for ordering the two elements (e.g., *Type X goes first/before Type Y*; cf. Suzuki et al. 2017). It is important to emphasize that it is currently unclear what kind of rule is in fact observed in *ABC-D* and that the syntactic nature of *ABC-D* thus remains unclear. To identify the rule, more data on sequences containing *ABC*, *D*, and other note combinations are needed. Nevertheless, because after hearing *D-ABC* about a fifth of the receivers still approached the caller but that there was only a very low average number of horizontal head scans in the sample (close to zero; Suzuki et al. 2016), it does appear that *D-ABC* is ungrammatical^4^. Specifically, it appears that (1) some of the receivers respond to *D-ABC* as if hearing only *D* (possibly because it precedes *ABC*) and that (2) most of the receivers do not respond to *D-ABC*. This lack of response to hearing *D-ABC* may be a result of inhibiting *ABC* after hearing *D*, presumably because it constitutes a rule violation. Future research on Japanese tit song will thus hopefully investigate (1) a possible reanalysis after hearing *ABC* in *D-ABC* (e.g., do some receivers initiate approaching the caller but subsequently abort, do they spend less time near the caller compared to when hearing *ABC-D*, etc.) and (2) the proportion of receivers which combine the approaching and scanning behaviors in response to *ABC-D* and *D-ABC*^5^.

This constitutes tentative evidence for semantic compositionality in Japanese tits because (1) the compositional message *ABC-D* consists of two elements for both of which it has been observed are meaningful when used individually and because (2) there seems to be a specific rule (something like *ABC* before *D*) governing how this particular compositional message should be coded (and decoded). Still, it is not entirely clear what kind of semantic relationship is established between *ABC* and *D* in the compositional message *ABC-D*. Tentatively, it might be suggested that the relationship is cumulatively conjunctive (i.e., “additive”, *and*-like),^6^ thus conveying a message like scan for danger and approach the caller. Another possibility is that the relationship here is not conjunctive but that *ABC* “modifies” *D*, with the compositional message thus conveying something like approach the caller with alertness. This is possible, yet appears less parsimonious in the light of the available data. To show that there is some kind of semantic modification going on here, (1) one would need to specify which behavioral components does alertness actually entail and are there variations in the presence or qualities of these components across different contexts (e.g., *ABC* used solely vs. *ABC* used in combination with other note combinations), (2) one would (ideally) observe *ABC* in combinations with other note combinations (apart from *D*) with the same or similar behavioral responses in the receivers, and (3) one would need to establish in the *ABC-D* example that the birds are “alert” only in association with approaching the caller.

### Semantic compositionality in non-human primates

Data on semantic compositionality in wild primates are lacking, leaving open the question of whether this phenomenon is a product of convergent evolution in the Japanese tits (and possibly other animals) or if it is part of the phylogenetic lineage leading to humans. In the last decade or so, it has been hotly debated whether the *pyow-hack* sequences in putty-nosed monkeys (*Cercopithecus nictitans*) (Arnold and Zuberbühler 2006a, b, 2008, 2012; Schlenker et al. 2016a, b) or the *krak-hok* sequences with the putative suffix *-oo* in Campbell’s monkeys (*Cercopithecus campbelli*) (Kuhn et al. 2018; Ouattara et al. 2009a; Rizzi 2016) can be regarded as semantically compositional but no firm conclusions have been drawn. Furthermore, there are currently no explicit indications in the literature to my knowledge that specifically wild apes (*Hominoidea*) might be capable of semantic compositionality (cf. Boesch and Crockford 2005; Leroux and Townsend 2020; Terrace et al. 1979). This is somewhat unexpected given that language experiments with captive apes have clearly demonstrated that they possess the capacity for at least some degree of semantic compositionality in both language production and language reception. For example, experiments with the female chimpanzee (*P. troglodytes*) Lana have demonstrated that language-taught captive chimpanzees can both understand and produce compositional messages in communication with humans. Lana was capable of meaningfully combining Yerkish lexigrams according to taught rules (such as word order), e.g. *Lana want eat bread,* which was a response to the (human) experimenter’s question*?* *Lana want what eat?* (Gill 1977; Rumbaugh 1977; Rumbaugh et al. 1973, 1974; Wallman 1992). Similarly, experiments with the male bonobo (*Pan paniscus*) Kanzi have shown that language-taught captive bonobos are also capable of semantic compositionality in both Yerkish lexigram production and English language reception (Savage-Rumbaugh et al. 1993; Segerdahl et al. 2005).

## Boesch’s (1991) Study

### Background

Boesch (1991) studied the behavior of a community of 80 wild chimpanzees (*P. troglodytes*) in a tropical forest within the Taï National Park, Côte d’Ivoire. The study reports on observations in the context of foraging activities that inherently involve travel. During foraging, the community would split “into a [sic] least three major parties that [could have] communicate[d] with one another by vocalising and drumming” (Boesch 1991: 81–82). A frequent behavior in the community was drumming, powerfully hitting and kicking of buttressed trees, typically preceded by loud pant-hooting. Thus, although drumming is a non-vocal communicational behavior, it is frequently accompanied by vocal behavior. Contrary to the sole drumming, the vocal behavior does not code parts of the message conveyed via drumming as a whole but serves, among others, caller identification.

The study focused on the drumming sequences by the alpha male Brutus, recorded between January 1983 and May 1984. Boesch developed a priori a six-category classification of the drumming sequences (Table 1). The categories differed in (1) the number of drumming events within a drumming sequence and (2) the number of trees used for drumming (i.e., whether the drumming events within the drumming sequence were performed on a single tree or whether they were distributed across two trees). In total, Boesch recorded 23 observations spread over a period of approximately 17 months. In the present paper, the categories *3A*† and *2A* + *2B*† were excluded from analyses since Boesch reported no such observations. Further, the category *4A* was also excluded from analyses given that there was only a single observation of *4A*. Thus, the present paper analyses 22 observations which can be classified into three categories: *2A*, *1A* + *1B*, and *1A* + *2B/2A* + *1B*.

**Table 1:** Classification of Brutus’s drumming communication

Category	Number of drumming events	Number of trees	Observations	Proposed semantic content
*2A*	2	1	8	resting period initiation
*1A* + *1B*	2	2	8	travel direction change
*3A*^†^	3	1	None	–
*1A* + *2B* or *2A* + *1B*	3	2	6	resting period initiation and travel direction change
*4A*	4	1	1	–
*2A* + *2B*^†^ or *1A* + *3B*^†^ or *3A* + *1B*^†^	4	2	None	–

Modified from Boesch (1991)

### Brutus’s drumming: unitary sequences

According to Boesch’s analysis, Brutus’s drumming sequences consisted of at least two drumming events within a maximally two-minute interval which conveyed information on either travel direction change, initiation of a resting period, or both. Only the drumming events in a quiet travel context were recorded as opposed to other contexts because the chimpanzees, including Brutus, drummed in various situations and frequently. Single drumming events by Brutus evoked “no special reaction” (Boesch 1991: 83) by others, indicating that sequences of two drumming events should be considered as unitary.

After Brutus would drum once on a tree and then drum once again on another tree within 2 minutes (*1A* *+* *1B*), the travel direction of the community would change. Boesch proposed the relationship between the form and meaning in *1A* *+* *1B* is relatively iconic, in the sense that other group individuals inferred the new travel direction by “mentally visualizing Brutus’s displacement between the two trees and then transposing it to their own direction of travel” (Boesch 1991: 83), implying other individuals extracted the message from auditory, not visual stimuli. We might hence assume that the drumming form *1A* *+* *1B* stands for something like travel direction change, while also intrinsically coding in which direction will the travel change. Semantically, this is very different from “words” observed in, for example, vervet monkeys (*Chlorocebus pygerythrus*) where distinct vocalizations denote object concepts, specifically, concepts for different types of predator and potentially hostile species: the leopard, martial eagle, African python, baboon, and unknown humans (Seyfarth et al. 1980a; Struhsaker 1967). In this sense, the semantic content of *1A* *+* *1B* might be more similar to the semantic content of the above-described Japanese tit song. More specifically, they are more abstract and seem to convey action-like or state-like (i.e., verb-like) meanings. Nevertheless, in both types of communication, imperative messages seem to be conveyed. In other words, in both types of communication the caller seems to (intentionally) “instruct” the receivers to change their behavior.

After Brutus would drum twice on the same tree (*2A*), the group’s activity would stop for approximately 60 min, when a lack of both vocalizations and bodily movement was observed among the individuals. Thus, *2A* was interpreted as conveying a (presumably imperative) message of resting period initiation. Unlike *1A* *+* *1B*, the relationship between form and meaning in *2A* does not appear iconic but arbitrary.

In one instance, Brutus was observed drumming four times on the same tree after which the group rested for two hours and 16 min. Because there was, unfortunately, only one such instance, there is not enough evidence for the existence of the form *4A* (or *2A* *+* *2A*) which would map to something like double rest. Still, if we were to assume that Brutus and other individuals also had *4A* in their vocabulary, the expression would strikingly resemble reduplicative expressions in human languages. In simple words, reduplication is the repetition of a word or its part for semantic or grammatical purposes. Often, reduplication is semantically associated with expressing larger quantities of something or meaning intensification. Examples include the Serbo-Croatian *glup-glup* ‘very stupid’ (from *glup* ‘stupid’), the Indonesian *pagipagi* ‘early morning’ (from *pagi* ‘morning’), or the Ilokano *kalkaldíng* ‘goats’ (from *kaldíng* ‘goat’) (Aronoff and Fudeman 2011; Marković 2013).

### Brutus’s drumming: semantically compositional sequences

Another sequence type was recorded with Brutus drumming twice on one tree and then once on another tree or drumming once on one and twice on another tree (*2A* +  *1B* or *1A* *+* *2B*). In both cases, the messages of resting period initiation and travel direction change were conveyed together, with the group first initiating the resting period and then continuing to travel in the new direction. According to available data, this constitutes evidence for semantic compositionality because (1) the sequences *2A* *+* *1B* and *1A* *+* *2B* are composed of parts of otherwise meaningful sequences and because (2) there is at least one rule for combining these two sequences (discussed below).

What kind of semantic relationship is established between parts of the unitary messages when they are combined into *2A* *+* *1B* or *1A* *+* *2B*?—Because Boesch reported no differences in the quality of the two behavioral responses between the unitary (i.e., *1A* *+* *1B* and *2A*) and putatively compositional messages, it seems most parsimonious that the relationship is cumulatively conjunctive (i.e., *and*-like). Still, in response to both *2A* *+* *1B* and *1A* *+* *2B*, the receivers would first rest and then continue to travel in the new direction, leaving open the possibility that the messages *2A* *+* *1B* and *1A* *+* *2B* also convey the order of the two events. It is also, however, possible that there is some cognitive constraint allowing the receivers to infer the order of events since resting has a typical duration (Boesch 1991), while the duration of travel may vary depending on different physiological, environmental, and other factors. The latter interpretation might be more parsimonious because there is no indication that the putative temporal meaning component is (overtly) expressed. For one thing, the putative temporal meaning component does not seem to be iconically coded by Brutus or inferred by the receivers from the order of the sequence’s elements given the seemingly free order of the elements. Thus, the relationship between the parts of the compositional messages appears cumulatively conjunctive, with possible addition of a temporal reference.

What kind of rule(s) govern(s) the combinatoriality of *1A* *+* *1B* and *2A*?—It is striking that this supposedly conjunctive compositional meaning is not expressed by simply juxtaposing the two unitary drumming sequences. This is the case in Japanese tits, where *ABC* and *D* are juxtaposed in a seemingly fixed order to convey a compositional message. If Brutus were simply juxtaposing the two drumming sequences, we would expect the form *3A* *+* *1B*† (or *1A* *+* *3B*†) for the compositional message. Compared to the form *3A* *+* *1B*† expected in a hypothetical juxtaposition context, the observed forms *2A* *+* *1B* and *1A* *+* *2B* appear to be structurally reduced by one drumming event. The observed signal reduction in Brutus’s compositional message is striking because (1) in previous studies with guenons (*Cercopithecus*) and Japanese tits the possibly/putatively compositional messages were composed of simply juxtaposed elements (with no apparent reduction or any kind of modification for that matter) and because (2) signal reduction is a fundamental feature of human language, detectable at various levels of linguistic communication. Signal reduction can be understood as an aspect of the economy principle of language which purports that in linguistic communication humans strive to exchange as much information as possible using as little effort as possible (Jaeger 2010; Jaeger and Buz 2018).

In Brutus’s case, the signal reduction appears to serve the formation of the sequence(s) using the elements *1A* *+* *1B* and *2A*. In human languages, signal reduction may also occur in cases where new words or expressions are formed. This is the case of blending, a word-formation phenomenon in which non-meaningful parts of typically two content words are combined to form another word (called a *blend)*. English is often cited as a language where blending is fairly productive with examples including *motel* (*motor* × *hotel*), *smog* (*smoke* × *fog*), *Brangelina* (*Brad Pitt* × *Angelina Jolie*), etc. (Aronoff and Fudeman 2011; Marković 2013). There is, however, at least one important difference between linguistic blending and the reduction observed in Brutus’s compositional message. Because in the form *1A* *+* *2B* the *1A* element can either be non-meaningful (as most of Brutus’s single drumming events, according to Boesch) or merely indicate that another drumming event on another tree will happen quickly which will provide information on a new travel direction, it can be said that *2B* codes information on both the travel direction change and resting period initiation. In blending, one part of the typically two parts of the blend cannot code both of the meanings of the blended words. Still, the ability of a specific element in the expression to be a realization of more elements (two, in Brutus’s case) together with the reduction of the form for the conjunctive message bare limited, yet remarkable resemblance to linguistic fusion. In fusion, two or more grammatical forms (specifically, morphs) are integrated into one which then realizes several grammatical meanings (i.e., morphemes). One example is the German *im* ‘approx. in the’ which is a fusion of *in* ‘approx. in’ and *dem* ‘approx. the’: If a German speaker wants to convey something like *I am in the supermarket.*, *in* (*in*) and *the* (*dem*) will be fused as in *Ich bin im Supermarkt.* (vs. *Ich bin in dem Supermarkt.*). French speakers will be familiar with the fusions *au* (*à* + *le*) or *du* (*de* + *le*) (Aronoff and Fudeman 2011; Marković 2013).

Unlike the Japanese tits, Brutus’s compositional message appears to have a flexible order of elements as both *2A* *+* *1B* and *1A* *+* *2B* appear grammatical. Still, the distribution of drumming events across the two trees appears fixed, given that Boesch only observed either one or two drumming events at a given tree and that the number of drumming events could not be the same for the two trees.

Despite all these observations from Boesch’s paper, we are still unable to identify the specific rule(s) for governing how the compositional message(s) should be coded. It can be inferred from the data that this rule encompasses signal reduction (by one drumming event), namely in such a way that one of the elements of the compositional sequence codes both resting period initiation and travel direction change. Unfortunately, future research on this community’s communication is practically impossible so we cannot hope that new data on communication in this community will eventually emerge. To come to some kind of a tentative inference about the rule(s) used in this community’s compositional messaging, we can ask ourselves whether there are similar expressions in human languages. I have already proposed that the two meanings in this communication system are verb-like in the sense that the forms denote an action (travel) and a state (rest) and that the message appears to express an imperative meaning. Thus, we might say that Brutus is combining two semantically imperative and verb-like messages. In fact, as language speakers, we should have no objections to the possibility of a similar utterance in English, e.g. *(Hey guys,) Let’s rest and then go that way!*. In other words, Brutus’s compositional message should seem natural to humans. Further, verb-verb constructions are found across languages. In Serbo-Croatian, for example, some simple verb-verb constructions can occur as idioms, e.g. *sjedi i plači* (lit. Sit and cry!, ‘approx. inconvenient situation; There is nothing you can do about it.’) or *povuci-potegni* (lit. Drag!-Pull!, ‘very difficult, painstaking, demanding’) (Jojić 2015). Similar examples can be found in Macedonian, e.g., *veži-dreši* (lit. Tie!-Untie!, ‘an ignorant person’) (Progovac 2015). Interestingly, the verbs in these constructions are all in the imperative form. In fact, simple noun–verb compounds in Serbo-Croatian are formed by joining a noun and a verb in the imperative form, e.g., *razbibriga* (lit. Break!-worry, ‘entertainment’), *ispičutura* (lit. Empty!-flask, ‘drunkard’), etc. (Progovac 2015, 2016). Similar to these verb-verb constructions are also short paratactic combinations of clauses,^7^ such as the English *Come one, come all.*, *Monkey see, monkey do.*, *Easy come, easy go.*, etc. (Progovac 2015, 2016) or the Serbo-Croatian *Došla, ošla.* (lit. [She] Came, [she] left., ‘approx. She was here for a short time.; She was unimportant.’) and *Sam pao, sam se ubio.* (lit. [You] Fell on your own, [you] died on your own., ‘approx. You are responsible for your own actions.’). Thus, Brutus’s compositional expressions appear structurally similar (to a degree) to verb-verb constructions and specifically paratactic combinations of two clauses.

### Arguments against semantic compositionality in Brutus’s communication

In Sect. 2.3. of the present paper, I proposed that Brutus’s communication displayed semantic compositionality based on the assumptions that (1) the unitary elements of the putatively semantically compositional message are meaningful when used individually and that (2) there is some kind of (not fully identified) rule governing how the compositional sequence should be formed. These arguments would typically suffice in the context of linguistics because in human linguistic communication, we can in most cases determine whether the receivers are producing specific behaviors in response to linguistic input from a speaker (as opposed to any other kind of stimulus). In wild animal communication research, however, it is often difficult to say whether the potential receivers are truly responding to a given communicative act. Relatively robust inferences and conclusions may be easier to draw from carefully controlled experimental studies. A famous example is the study on vervet monkeys by Seyfarth et al. (1980a) (mentioned above). In order to test the hypothesis that specific alarm calls denote concepts as human words do, the authors hid loudspeakers from which they played recordings of the different alarm calls (in the absence of the actual predators). In response to the recordings, the vervet monkeys engaged in the predator-specific behaviors. This constituted evidence that the alarm calls evoke concepts of specific predators in the semantic memory of the receivers as it was clear that the receivers did not change their behavior because they saw (or otherwise perceived) the predators (either independently or cued by other callers). Thus, to further argue that Brutus’s putatively compositional messages are in fact semantically compositional (and that the community’s communication as a whole is semantic), we must show that other individuals in the community responded to the auditory stimuli and their proposed semantic content and not, for example, by visually observing Brutus’s behavior after the drumming sequences. According to the latter hypothesis, the different drumming sequences would presumably act as cues for locating Brutus and initiating visual examination of his behavior in other individuals.

Unfortunately, Boesch does not explicate in his paper that other individuals were not visually examining Brutus’s behavior after he had drummed. Despite this, it seems highly unlikely to me that the visual hypothesis is true. There is an abundance of indications in Boesch’s paper that other individuals were in fact responding to the proposed meanings conveyed by the drumming sequences. Firstly, visual contact between individuals was limited, while visual contact between individuals of different foraging parties of the same community appears to have been extremely limited or non-existent: “[The] receivers [were] often out of visual contact with Brutus” (Boesch 1991: 86). At the same time, Boesch (1991: 81) writes that the individuals were “permanently in auditory contact with the majority (72%) of the community (80 chimpanzees)”. The lack of visual contact is due to the low visibility in the Taï rainforest with the “visibility on the ground rarely exceeding 20 m” (Boesch 1991: 81). In Example 1, Boesch (1991: 83) describes a situation in which Brutus drummed *1A* *+* *1B*. Boesch writes that one “noisy” foraging party was moving about 500 m in front of Brutus when Brutus drummed, after which Brutus “silently and alone” and “in a leisurely way” continued his travel. Boesch’s description of Brutus’s behavior after drumming suggests he was not attracting visual or other attention, while the distance between Brutus and the observed party (and presumably other parties) suggests that other individuals could not have simply visually examined Brutus’s behavior after drumming. In this context, it is also important to note that chimpanzee individuals have characteristic call styles. There also appears to have been little or no communication between individuals after Brutus’s drumming: “[N]ormally the group would follow Brutus’ proposals without any vocalisation, with no sound being made for the next one or more kilometers” (Boesch 1991: 86). Thus, we can also exclude the hypothesis that those individuals who had visual contact with Brutus somehow communicated this to other individuals. Secondly, the visual hypothesis does not explain why Brutus produced different drumming sequences before initiating the specific behaviors. Boesch (1991: 84) writes that “[n]o disagreement between [his] predictions and the chimpanzee responses occured [sic]”. It is unclear why Brutus would have produced specific drumming sequences if he were merely attracting visual attention from other individuals.

There is another possible issue in interpreting Brutus’s communication as semantically compositional. In speech, elements of semantically compositional messages are not normally separated by longer intervals of silence. The existence of these intervals has been used by some as an argument that the putatively compositional expressions by Campbell’s monkeys are not semantically compositional^8^. While I am unable to resolve this issue, I would like to point out that in some specific but normal contexts, humans can also be interpreted as displaying longer intervals of silence between elements of one compositional message. For example, during athletic running competitions, the message of race initiation is often conveyed by expressing something like *On your marks!–Set!–Go!*. Conventionally, if any of the three elements is missing, a race cannot be initiated. Thus, it can be said that *On your marks!–Set!–Go!* is a compositional message. At the 2016 Olympics, the interval between *On your marks!* and *Ready!* (equivalent to *Set!*) during the men’s 100-m final race was approx. 37 s. Thus, the presence of intervals of silence between elements of a supposed compositional expression in Brutus is not necessarily an argument that Brutus is not exhibiting semantic compositionality.

## Final arguments

In the present reanalysis of Boesch’s (1991) descriptive study, I proposed that a community of wild chimpanzees (*P. troglodytes*) in the Taï National Park displayed semantic compositionality in their communication. More specifically, the alpha male Brutus produced semantically compositional expressions and this compositional message was received by other individuals of the community. I have proposed that there is evidence for semantic compositionality in Brutus’s communication given that (1) the elements of the compositional expression are meaningful when used individually (travel direction change and resting period initiation) and (2) because there is some kind of rule (at least one) for combining these elements into the compositional expression. This is the first paper to my knowledge proposing that wild apes are exhibiting semantic compositionality in their communication. Thus far, the only strong evidence for semantic compositionality in wild animal communication has been found for a songbird (Suzuki et al. 2016). The presence of semantic compositionality in wild chimpanzee communication suggests that this communicational feature was possibly also present in the last common ancestor of humans and chimpanzees and, by extension, hominins predating *Homo sapiens*.

In describing the semantic relationship which is established between the elements of the compositional message, I have proposed that the relationship is cumulatively conjunctive, i.e., *and*-like.

Due to a lack of data on other kinds of drumming sequences and their potential meanings, it is difficult to identify the rule(s) used for combining the elements in Brutus’s compositional expression (similarly to the situation with Japanese tits). However, Brutus’s compositional expression clearly displays signal reduction and the capacity of one element of the expression to code the meanings of both of the elements in the compositional expression. In this regard, Brutus’s compositional expression is very different from the one reported for Japanese tits where two elements are simply juxtaposed without apparent reduction or other modification. Remarkably, these features of Brutus’s compositional expression bear some resemblance to specific linguistic phenomena such as fusion and blending. Further, Brutus’s compositional expression shows resemblance to certain verb-verb constructions in which the verbs are in the imperative form, as well as short paratactic combinations of two clauses.

Progovac (2015, 2016) has introspectively discussed at length her hypothesis that such short paratactic combinations of clauses, as well as simple verb-verb constructions and noun–verb compounds in which the verb is in the imperative form are a kind of a “language fossil”, a structural remnant of the early stages of language evolution present in all languages, due to their relative syntactic unspecificity and the need to combine only two words (so-called *two-slot grammar*). Specifically, Progovac believes that such linguistic phenomena form a kind of “protosyntax” which was present at least from *Homo heidelbergensis* (i.e., the phylogenetically intermediate species between *Homo erectus* and anatomically modern humans). Similar, yet less elaborate proposals have been made by others as well (e.g., Barham and Everett 2020; Benítez-Burraco and Progovac 2020; Botha 2020; Dediu and Levinson 2013, 2014, 2018; Everett 2017; Gabrić 2019, 2021a, b, c; Gabrić et al. 2018, 2021; Gil 2008, 2009; Michlich 2018). The current reinterpretation of Brutus’s communication suggests the possibility that such or similar communicational behaviors might have been present before the human-chimpanzee split and, by extension, in hominins predating *Homo sapiens*, although not necessarily in the vocal modality. It is also interesting that the semantic compositionality of Brutus’s communication was observed in a foraging context, as previous discussions have proposed that the complexity of some hunting behaviors in extinct hominins might be an indicator of linguistic communication in these species/populations (e.g., Botha 2020), possibly suggesting an association between subsistence strategies and food acquisition, and the emergence of more complex communication.

## Conclusion

A reanalysis of Boesch’s (1991) study of a chimpanzee community in the Taï National Park (Côte d’Ivoire) revealed that the alpha male Brutus produced semantically compositional combined messages of travel direction change and resting period initiation. Unlike the Japanese tits, the elements of the compositional expression were not simply juxtaposed but displayed structural reduction, while one of the two elements in the expression coded the meanings of both elements. These processes show relative resemblance to blending and fusion in human languages. Also unlike the tits, the elements of the compositional expression did not have a fixed order, although there was a fixed distribution of drumming events across the trees used for drumming. Because the elements of the expression appear to carry verb-like meanings, the compositional expression also resembles simple verb-verb constructions and short paratactic combinations of two clauses found across languages. In conclusion, the reanalysis suggests that semantic compositionality and phenomena resembling paratactic combinations of two clauses might have been present in the communication of the last common ancestor of chimpanzees and humans, although not necessarily in the vocal modality.

## Data Availability

Not applicable.

## Footnotes

1 In the present paper, I use the term *rule* with the meaning *observed pattern of communication*. The term *rule* does not imply that the communicator is aware of the “rules” they are using or has other conscious access to the “rule(s)”. The term *rule* is often used in linguistics in the context of grammar/syntax.

2 In some schools of thought, semantic compositionality is a highly controversial notion, while according to some, the phenomenon does not exist but is simply a construct of linguistic theorizing (see Pagin and Westerståhl 2019; Pelletier 1994, 2016; Sweetser 1999).

3 Small caps are used for concepts (meanings) (cf. Hurford 2007).

4 Grammaticality is a graded phenomenon, i.e., some expressions may be more grammatical than others, e.g. *He behind the box.* vs. *He the behind box.* Ungrammaticality does not (necessarily) imply unintelligibility (Ambridge 2014; Myers 2017). An expression may be grammatically acceptable, yet semantically unacceptable, e.g., *The pizza was too hot to cry.* or *My mom made a telephone cake*.

5 Unfortunately, Suzuki et al. (2016) calculated the rates of the two behaviors separately and did not report how many receivers displayed either only one or both of the behaviors.

6 Semantically, conjunction can be understood as joining at least two meaningful structures in some kind of relationship. Cumulative conjunction is a type of conjunction in which at least two meaningful structures are paired/combined (e.g., *bread and milk*). The term *coordination* is used when the conjunctive meaning is (overtly) expressed, for example, by using the coordinator (i.e., coordinating conjunction) *and* (e.g., *They sang, danced, and drank.* vs. *They sang, danced, drank.*) (cf. Jasinskaja and Zeevat 2009; Mitrović and Sauerland 2014; Saeed 2015; Zeevat and Jasinskaja 2007). This is because (cumulative) conjunction does not have to be (overtly) expressed (e.g., *It was the best of times, it was the worst of times*; Trask 1993). Problematically, *conjunction* may refer to different phenomena in the linguistic literature, not necessarily related to semantic conjunction (for clarification, see Börjars 2015; Crystal 2008; Trask and Stockwell 2007).

7 In paratactic combinations of clauses, the clauses are juxtaposed without the use of any connecting device. Parataxis is opposed to hypotaxis (Trask 1993).

8 “The 20-s delay between the booms and the alarm call is problematic, as it does not suggest composition of a unitary message. One would expect a unitary communicative utterance consisting of several parts to be produced with little or no delay between the parts (unlike the slow stately progress of whale songs)” (Hurford 2012: 15).
